# Double-labelling immunohistochemistry for MGMT and a “cocktail” of non-tumourous elements is a reliable, quick and easy technique for inferring methylation status in glioblastomas and other primary brain tumours

**DOI:** 10.1186/2051-5960-1-22

**Published:** 2013-06-10

**Authors:** Elinor Burke, Mariana Grobler, Kay Elderfield, Frances Bond, Matthew Crocker, Rohan Taylor, Leslie R Bridges

**Affiliations:** 1 Cellular Pathology, St George’s Hospital, Blackshaw Road, London SW17 0QT, UK; 2 Molecular Genetics, St George’s Hospital, Blackshaw Road, London SW17 0QT, UK; 3 Neurosurgery, St George’s Hospital, Blackshaw Road, London SW17 0QT, UK

**Keywords:** MGMT, Methylation, Glioblastoma, Glioma, Immunohistochemistry, Double-labelling, Cocktail, MLPA, Biomarker

## Abstract

**Background:**

Our aim was to develop a new protocol for MGMT immunohistochemistry with good agreement between observers and good correlation with molecular genetic tests of tumour methylation. We examined 40 primary brain tumours (30 glioblastomas and 10 oligodendroglial tumours) with our new technique, namely double-labelling immunohistochemistry for MGMT and a "cocktail" of non-tumour antigens (CD34, CD45 and CD68). We compared the results with single-labelling immunohistochemistry for MGMT and methylation-specific multiplex ligation-dependent probe amplification (MS-MLPA, a recognised molecular genetic technique which we applied as the gold-standard for the methylation status).

**Results:**

Double-labelling immunohistochemistry for MGMT produced a visual separation of tumourous and non-tumourous elements on the same histological slide, making it quick and easy to determine whether tumour cell nuclei were MGMT-positive or MGMT-negative (and thereby infer the methylation status of the tumour). We found good agreement between observers (kappa 0.76) and within observer (kappa 0.84). Furthermore, double-labelling showed good specificity (80%), sensitivity (73.33%), positive predictive value (PPV, 83.33%) and negative predictive value (NPV, 68.75%) compared to MS-MLPA. Double-labelling was quicker and easier to assess than single-labelling and it outperformed quantitative computerised image analysis of MGMT single-labelling in terms of sensitivity, specificity, PPV and NPV.

**Conclusions:**

Double-labelling immunohistochemistry for MGMT and a cocktail of non-tumourous elements provides a "one look" method for determining whether tumour cell nuclei are MGMT-positive or MGMT-negative. This can be used to infer the methylation status of the tumour. There is good observer agreement and good specificity, sensitivity, PPV and NPV compared to a molecular gold-standard.

## Background

Clinical trials have shown that determination of the methylation status of glioblastomas is an important predictor of outcome and response to treatment [1]–[4]. A range of molecular genetic techniques are available for determining methylation status in tumours [5]. However, these are generally expensive and somewhat slow in application. It would certainly be of great help if immunostaining were available to infer the methylation status of the tumour. In fact, immunostaining for MGMT, on the face of it, should provide an answer. Antibodies against MGMT are readily available which stain tumour cell nuclei positive in unmethylated tumours and negative in methylated tumours (this opposite relationship between MGMT staining and methylation status is due to the fact that methylation of the MGMT gene switches off production of the MGMT protein) [6].

However, there is a major problem in interpretation of MGMT immunostaining in brain tumours due to the fact that normal non-tumourous elements within the tumour (including endothelial cells, lymphocytes and macrophages) normally express MGMT [6]–[8]. This makes it difficult to determine whether any MGMT positivity within the tumour is due to tumour cells or non-tumour cells. Not surprisingly, the literature therefore contains much controversy about the value or otherwise of MGMT immunostaining as a clinical biomarker in brain tumours (please see Table 1).

**Table 1:** Previous studies

**Author and year**	**Tumour type**	**Cut off value for IHC**	**Low MGMT protein expression predictive of:**		**MGMT IHC compared to molecular gold standard**	**Molecular test**	**Correlation between IHC and molecular test**
			**Progression free survival** (**PFS**)	**Overall survival** (**OS**)			
Nakasu *et al*. 2004 [7]	69 high-grade gliomas (grades III and IV)	10%	-	Yes	No	-	-
Brell *et al*. 2005 [11]	93 anaplastic gliomas: 75 AAs and 18 with oligodendroglial component	5%	No	Yes	Yes	MS-PCR	No
Chinot *et al*. 2007 [21]	28 GBMs	35%	Yes	Yes	No	-	-
Capper *et al*. 2008 [22]	75 primary GBMs	15%	Yes – median survival		No	-	-
Preusser *et al*. 2008 [6]	164 GBMs	10%	-	No	Yes	MS-PCR	Poor agreement
Rodriguez *et al*. 2008 [23]	50 GBMs	10%	No	No	Yes	MS-PCR	No
Karayan-Tapon *et al*. 2010 [24]	81 GBMs	15%	No	No	Yes	MS-PCR	-
						SQ-PCR	No
						Pyrosequencing	Yes
						Q-RT-PCR	Yes
Quillien *et al*. 2012 [5]	100 GBMs	23%	Yes	Yes	No	-	-

The table shows previous studies where MGMT IHC has been performed. The cut-off value was the percentage of MGMT-positive tumour cell nuclei (assessed by eye). The correlation of IHC with progression-free survival (PFS) and overall survival (OS) and comparison with a molecular gold standard (where applied) are also shown.

Abbreviations: *AA* anaplastic astrocytoma, *GBM* glioblastoma multiforme, *MS*-*PCR* methylation-specific polymerase chain reaction, *Q*-*RT*-*PCR* quantitative real-time polymerase chain reaction, *SQ*-*PCR* semi-quantitative polymerase chain reaction.

For example, Preusser *et al*. [6] state that observer variability and lack of association with patient survival impedes the use of MGMT immunostaining in glioblastomas. On the other hand, Watanabe *et al*. [9] (on the basis of eight years clinical experience of MGMT immunostaining, using serial sections stained for non-tumour markers such as CD45 and CD68 and a careful algorithmic approach) consider that MGMT immunohistochemistry correlates with outcomes in patients with glioblastomas.

Although the use of serial sections stained with non-tumour markers for comparison with sections stained for MGMT is undoubtedly helpful, it is not always easy to compare cellular identities between serial sections. To some extent it is possible to identify tumour cells in the MGMT-stained section directly (for example, when tumour cell nuclei are very large and bizarre). However, reactive elements within tumours may themselves develop a degree of nuclear enlargement and irregularity which makes distinction from tumour cells impossible at times.

Double-labelling immunohistochemistry is an established technique which is now available on some automated immunohistochemistry platforms such as the Leica platform in use in our laboratory. This means that double-labelling protocols can readily be incorporated into the routine work of the laboratory, with much the same turnaround time as standard immunohistochemistry and at little extra cost.

We have used such a platform to develop a novel technique, namely double-labelling immunostaining of MGMT and a "cocktail" of non-tumour antigens (CD34, CD45 and CD68). Our aim was to produce a visual separation of tumour cells and non-tumour cell elements on the same histological slide and thereby greatly simplify the assessment of MGMT immunostaining in tumour cell nuclei.

## Results

**Figure 1:**
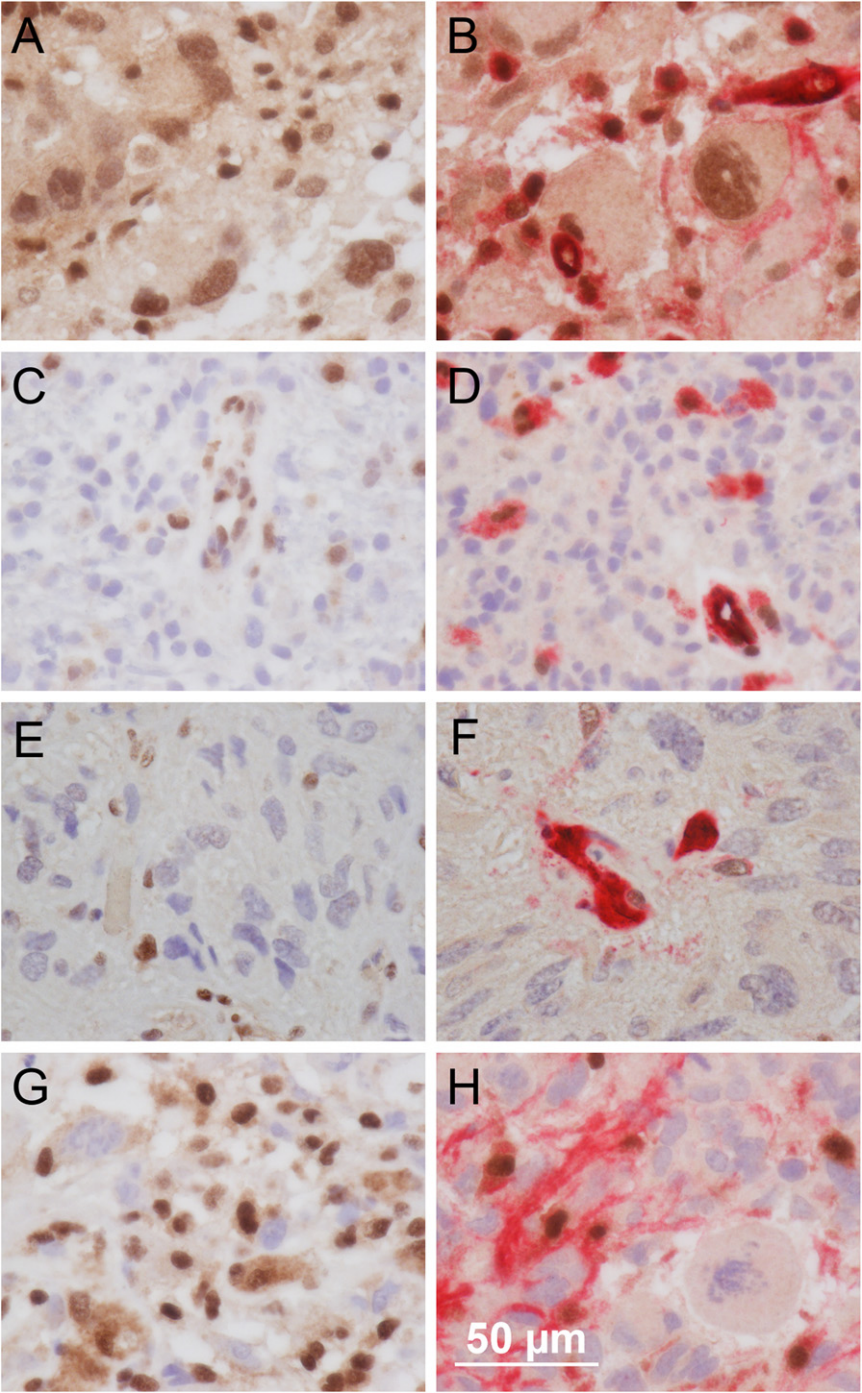
**Photomicrographs comparing single**-**labelling and double**-**labelling for MGMT.** This figure shows the advantage of double-immunolabelling over single-immunolabelling in the interpretation of MGMT immunostaining. Four glioblastomas are shown, one per row, with the single-labelling for MGMT on the left and double-labelling for MGMT and "cocktail" (combined CD34, CD45 and CD68) on the right. All images were photographed at an objective lens magnification of 80x (scale bar = 50 microns). For all images, nuclear immunostaining for MGMT is seen as brown due to the DAB-peroxidase product. For images on the right, cytoplasmic staining for CD34, CD45 and CD68 is seen as red due to the Fast Red-Alkaline Phosphatase product used in the double-labelling system. The counterstain is haematoxylin (nuclei unstained for MGMT appear blue). The case in row 1 (case 25 in Table 2) was unmethylated by MLPA. Double-labelling (**B**) is more informative than single-labelling (**A**) because it highlights the cytoplasm of the non-tumourous elements (endothelial cells, lymphocytes and macrophages) as red. The remaining cells can therefore be positively identified as tumour cells and the presence of undoubted nuclear immunostaining for MGMT in these tumour cells correctly indicates an unmethylated status. The case in row 2 (**C** and **D**; case 39 in Table 2) was extensively methylated by MLPA. The case was easily assessed as MGMT-negative (methylated) on double-labelling (**D**). The case in row 3 (**E** and **F**; case 18 in Table 2) illustrates the issue of "equivocal" staining as seen a number of cases (asterisked in Table 2). Please see Results for further commentary on this phenomenon. The case in row 4 (case 22 in Table 2) illustrates a situation where single-labelling (**G**) shows a high labelling index for MGMT due to a high content of non-tumourous cells (endothelial cells, lymphocytes and macrophages). Double-labelling (**H**) provides an easy "one-look" diagnosis of MGMT-negative (methylated).

A comparison of the results for double-labelling versus single-labelling and MLPA (the gold standard) is shown in Table 2. Some representative examples of single-labelling and double-labelling are shown in Figure 1.

**Table 2:** Cases

**case ID**	**type**	**LI**	**SL**	**MLPA**	**MLPA 0**-**3**	**MLPA** >**0**.**25**	**DL**
1	oligo	7.567		1	0.6275	2	1	1
2	oligo	13.700		1	0.675	2	1	1
3	oligo	12.600		1	0.8075	3	1	1
4	oligo	24.667		1	0.4725	1	1	1
5	oligo	17.100		1	0.8525	3	1	1
6	oligo	13.600		1	0.8025	3	1	1
7	oligo	19.567		1	0.6425	2	1	1
8	oligo	14.267		1	0.6925	2	1	1
9	oligo	8.800		1	0.6525	2	1	1
10	oligo	26.133		1	0.5925	2	1	*1
11	gbm	56.367		0	0.1125	0	0	0
12	gbm	19.167		1	0.8475	3	1	1
13	gbm	84.400		0	0.09	0	0	0
14	gbm	17.967		1	0.2625	1	1	*1
15	gbm	57.700		0	0.255	1	1	0
16	gbm	89.900		0	0.4075	1	1	0
17	gbm	72.267		0	0.2075	0	0	0
18	gbm	19.533		1	0.3525	1	1	*1
19	gbm	54.133		0	0.42	1	1	*1
20	gbm	53.900		0	0.1375	0	0	0
21	gbm	21.967		1	0.485	1	1	1
22	gbm	54.167		0	0.5475	2	1	1
23	gbm	33.000		1	0.085	0	0	0
24	gbm	76.300		0	0.2725	1	1	0
25	gbm	96.733		0	0.24	0	0	0
26	gbm	49.567		1	0.4275	1	1	1
27	gbm	43.967		1	0.1375	0	0	*1
28	gbm	40.533		1	0.1025	0	0	0
29	gbm	7.233		1	0.1275	0	0	1
30	gbm	39.067		1	0.5125	2	1	1
31	gbm	25.833		1	0.2075	0	0	*1
32	gbm	33.900		1	0.13	0	0	*1
33	gbm	28.000		1	0.365	1	1	0
34	gbm	45.000		1	0.275	1	1	1
35	gbm	84.100		0	0.205	0	0	0
36	gbm	85.100		0	0.2975	1	1	0
37	gbm	64.933		0	0.11	0	0	0
38	gbm	53.400		0	0.17	0	0	0
39	gbm	6.800		1	0.825	3	1	1
40	gbm	22.500		1	0.2425	0	0	0

Case ID (identification) is the case number we assigned in this study. Tumour types were oligodendroglial tumours (oligo) and glioblastomas (gbm). The oligodendroglial tumours were oligodendroglioma WHO grade II (cases 4, 5 and 6), anaplastic oligodendroglioma WHO grade III (cases 1, 2, 3, 7 and 9), oligoastrocytoma WHO grade II (case 10) and anaplastic oligoastrocytoma WHO grade III (case 8). The glioblastomas were all glioblastoma WHO grade IV except cases 13 and 18 which were glioblastoma with oligodendroglioma component WHO grade IV. LI is the nuclear labelling index for single-labelled MGMT as determined by computerised image analysis. SL is the binarised score for the single-labelling (0 = LI > 50%, 1 = LI from 0 to 50%). MLPA is the MLPA ratio (please see text). MLPA 0–3 is the score for MLPA according to cut-offs for the MLPA ratio provided by Jeuken et al. 2007 [10] (0 = unmethylated, 1 = mildly methylated, 2 = moderately methylated and 3 = extensively methylated). MLPA > 0.25 is the binarised methylation score for MLPA (0 = unmethylated, 1 =methylated). DL is the binarised score for double labelling (0 = mainly MGMT-positive in tumour cells, 1 = mainly MGMT-negative in tumour cells). The asterisked cases were initially assigned a score of 1 in the tripartite system (please see text for explanation).

### Agreement in assessment of the double-labelling

There was good inter-observer and intra-observer agreement in the assessment of the double-labelling. For the three independent "blind" observers (EB, LB and KE) all appraisers’ assessments agreed with each other for 31/40 (77.50%) of the cases. The Fleiss’ kappa statistic was 0.76 and Kendall’s coefficient of concordance for ordinal scores was 0.93 indicating a good level of agreement. The intra-observer agreement was 36/40 (90%) of cases matched with a kappa of 0.84 and a Kendall of 0.97.

### Sensitivity, specificity, positive predictive value and negative predictive value of double-labelling

The results of the neuropathologist (LB) were compared with the gold standard, MLPA. For this, cases assessed as largely MGMT-negative were considered methylated (i.e. test result positive) and cases assessed as largely MGMT-positive were considered unmethylated (i.e. test result negative). For MLPA we use the cut-off used by Jeuken *et al*. [10]. According to this, all cases with an MLPA ratio greater than 0.25 are at least mildly methylated (i.e. true positives) and cases with an MLPA ratio of between 0 and 0.25 are unmethylated (i.e. true negatives).

Of the 40 cases, there were 11 negative matches, 20 positive matches, 4 false-positives and 5 false-negatives. The test results showed significant association (chi-square, p = 0.001) between the double-labelling and MLPA results. Sensitivity was 80%, specificity 73.33%, positive predictive value (PPV) 83.33% and negative predictive value (NPV) 68.75%. For the 30 glioblastomas apart, sensitivity was 66.7%, specificity 73.33%, PPV 71.43% and NPV 68.75%. All 10 of the oligodendroglial tumours were correctly called. These values indicate a good all-round performance by the double-labelling.

The other assessors’ results were also compared with MLPA. The results for specificity, sensitivity, PPV and NPV were 88%, 46.67%, 73.33% and 70% for KE and 88%, 40%, 70.97% and 66.67% for EB. The results are similar to LB’s apart from sensitivity which scored less well for these assessors. This may reflect the particular approach of the assessors; the neuropathologist appears to have been more willing to ignore low levels of MGMT staining than the two laboratory scientists. In this paper we have carried forward the neuropathologist’s scores on the basis that the pathologist carries out the everyday reporting in practice.

### False-negatives and false-positives

Of the 5 false-negatives it is interesting to note that all were only mildly methylated on MLPA. The slides were reviewed and all 5 cases were confirmed as showing undoubted MGMT-positivity suggestive of an unmethylated status. It is therefore of interest that although these cases were false negatives, the result of MLPA was only mild methylation – the closest category to unmethylated. Further clinicopathological correlation may help develop a better understanding of the significance of the "mildly" methylated category on MLPA.

The slides of the 4 false-positives were also reviewed. One case was undoubtedly MGMT-negative suggestive of a methylated status but MLPA was unmethylated. The other 3 cases showed equivocal MGMT-immunostaining and we had chosen by convention to call these MGMT-negative (please see below).

### Equivocal MGMT-immunostaining

Table 2 shows 7/40 cases (asterisked) with equivocal (focal or faint) MGMT-immunostaining of tumour cell nuclei on double-labelling. For illustrations of equivocal staining please see Figure 1 (E and F). These cases were difficult to classify as MGMT-negative (methylated) or MGMT-positive (unmethylated). We therefore decided on a convention by which such cases were interpreted as MGMT-negative (suggestive of a methylated status). Of the 7 such cases, 4 cases (3 glioblastomas and 1 oligodendroglial tumour) turned out to be correctly thus-called (i.e. they were methylated by MLPA). Three cases (all glioblastomas) were incorrectly called (i.e. they were unmethylated).

Since there is no clear indication at present from these figures as to how the equivocal cases should be assessed (roughly equal numbers were methylated and unmethylated) we suggest - until more data is available - treating these cases as MGMT-negative (suggestive of a methylated status) as we have here. It may be worth noting in the pathology report that such cases - in particular - are liable to a change in status when molecular testing is through. This approach would seem most consistent with the default clinical position whereby patients with glioblastomas in which the methylation status is unknown are managed as if the tumour were methylated. We are gathering further data on the issue of equivocal staining which, as indicated, accounts for about a fifth of our assessments.

### Comparison with single-labelling

As mentioned, Figure 1 shows how quantitative assessment of double-labelling is quick and easy compared to single-labelling. We wondered how the automated assessment of the labelling index for MGMT on single-labelling performed against the qualitative assessment on double-labelling. For this we set a convenient cut-off of 50% for the labelling index (close to the actual median of 47.28 for the 40 cases in our series). Cases with a labelling index of up to 50% were considered methylated (i.e. a positive test result) and cases with a labelling index greater than 50% were considered unmethylated (i.e. a negative test result) and this was compared with MLPA using the 0.25 cut-off of Jeuken *et al*. [10] as previously detailed. Please note that our choice of a median cut-off for the single-labelled MGMT labelling index may at first sight appear to be a departure from previous studies (e.g. Preusser *et al*. [6]). Generally these other studies have used assessment "by eye” and mentally subtracted tumour-only elements whereas our data is truly quantitative and applied to both tumourous and non-tumourous elements since the machine cannot separate the two. We feel that the median cut-off is appropriate in our context.

For all cases (n = 40) for the single-labelling MGMT labelling index versus MLPA sensitivity was 76%, specificity 55.33%, PPV 73.08% and NPV 57.14%. For the glioblastomas (n = 30) sensitivity was 60%, specificity 53.33%, PPV 56.25% and NPV 57.14%. Compared to double-labelling these results for quantitative single-labelling are not as good and there are also other disadvantages compared to double-labelling (please see Discussion).

**Figure 2:**
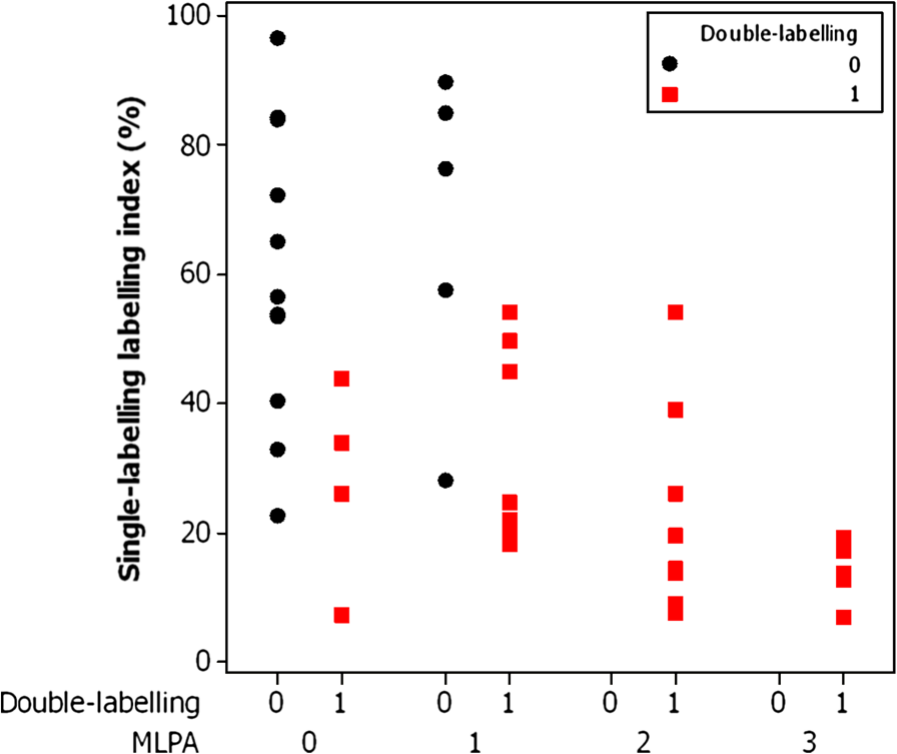
**Chart of MGMT single**-**labelling**, **MGMT double**-**labelling and MLPA status.** The chart shows the labelling index for MGMT from the single-labelled slides on the Y-axis for the 40 cases plotted against the results of double-labelling and MLPA status. The 4 cases that were false-positive by double-labelling are seen in the second column from the left. All of these cases have a labelling index of less than 50% by single-labelling suggestive of a methylated status. The results of double-labelling and single-labelling are therefore concordant and combined analysis would not have avoided calling these cases (falsely) positive. The 5 cases that were false-negative by double-labelling are seen in the third column from the left. Of these, 4 have a labelling index of greater than 50% by single-labelling suggestive of an unmethylated status. The results of double-labelling and single-labelling of these 4 cases are therefore concordant and combined analysis would not have avoided calling these (falsely) negative. Only one of the 5 false-negative cases has a labelling index of less than 50% by single-labelling suggestive of a methylated status. In this case the labelling index might have been helpful in avoiding calling this (falsely) negative on double-labelling. Overall however the extra effort involved in measuring the labelling index by automated quantitative image analysis on the single-labelled slides may not be worthwhile, particularly in labs not set up for image analysis.

We then wondered whether the single-labelling and double-labelling could be combined in some way to give a better result and the analysis is shown in Figure 2.

## Discussion

We have developed a novel protocol for the analysis of MGMT-immunostaining using double-labelling. The aim of our approach is to simplify the assessment of MGMT-immunostaining by creating a visual separation of the tumourous and non-tumourous elements. We did this by using the usual brown chromogen (DAB-peroxidase) for nuclear MGMT staining and a red chromogen (Fast Red-Alkaline Phosphatase) for a “cocktail" of non-tumourous cytoplasmic elements. The cocktail is composed of CD34 (for endothelium), CD45 (for lymphocytes) and CD68 (for macrophages). The end result was a histology slide in which we could easily distinguish between tumourous and non-tumourous components and thereby quickly come to a conclusion as to whether the tumour cell nuclei were MGMT-positive or MGMT-negative (and thence to a conclusion as to their likely methylation status).

We found good agreement between observers (kappa 0.76) and within observer (kappa 0.84). Furthermore, double-labelling showed good specificity (80%), sensitivity (73.33%), positive predictive value (83.33%) and negative predictive value (68.75%) compared to MLPA (the gold-standard).

Compared to qualitative assessment of single-labelling MGMT-immunostaining, we found double-labelling to be much quicker and easier to assess (Figure 1). Furthermore, double-labelling outperformed even quantitative computerised image analysis of MGMT single-labelling in terms of sensitivity, specificity, PPV and NPV. Furthermore, we did not find that much benefit would accrue from combining the results of double-labelling with quantitative single-labelling. Since quantitation is somewhat time-consuming we would therefore not necessarily recommend using both double-labelling with quantitative single-labelling on a routine basis: we consider double-labelling alone to be sufficient for everyday purposes.

We are aware that the double-labelling as with any test is not perfect and that certain cautions are required in interpretation. The cocktail of three reagents (CD34, CD45 and CD68) should stain a good proportion of non-tumourous elements but not necessarily all. For example, there may also be non-neoplastic astrocytes and oligodendrocytes which are not identified by the cocktail. For this reason we would recommend that interpretation of the double-labelling be focussed on areas of tumour proper and caution exercised in areas where tumour is infiltrating largely normal tissues. We are also aware that CD34 occasionally stains glioblastoma tumour cells and in such situations tumour cells will be stained red by the red chromagen and will thus be excluded from analysis. However, we feel the CD34 staining by glioblastomas cells is usually just focal so that it should be possible in most cases to find interpretable areas in the slide.

In our own current practice, we carry out double-labelling for MGMT and "cocktail" (CD34, CD45 and CD68) to come to an initial conclusion as to the likely methylation status of the tumour. Our report contains a caveat that this result may be overturned by MLPA. Nevertheless, because of the quicker turnaround time of immunohistochemistry compared to molecular genetic testing, the clinicians find this helpful with their initial management pending the definitive result from MLPA. At present, all cases are followed up with MLPA for definitive diagnosis of methylation status. It may be possible in time, when more information has been gathered about double-labelling, to phase out some of the MLPA testing.

In future studies we would like to compare the results of double-labelling for MGMT and a “cocktail” of non-tumourous elements to clinical outcomes. Quantitation of MGMT expression in tumour cells in double-labelled images will require special thresholding protocols but should provide useful comparison with the methylation ratios obtained from MLPA. Although MLPA is a relatively new technique for assessment of methylation status, in this regard it has the advantage of providing a quantitative result. The double-labelling-cocktail method may also be useful in the study of heterogeneity of MGMT expression in brain tumours. A number of recent studies [11]–[18] have raised the question of whether or not a strict correlation should really be expected between MGMT staining and methylation status. For example, Kreth and colleagues [12] found that patients with discordant results for MGMT expression (determined by mRNA expression) and methylation status did worse than concordant counterparts. A molecular basis for MGMT expression independent of methylation may apply in some situations [13]. Our double-labelling technique offers a possible new approach to investigate such questions.

## Conclusions

We have developed a novel method for double-labelling for MGMT, which is quick and easy to apply and might readily be subsumed into routine reporting for glioblastomas (and other tumours). There is good inter-observer and intra-observer agreement and, compared to a molecular genetic gold-standard, the method yields good sensitivity, specificity, positive predictive value and negative predictive value as a predictor of methylation status.

## Methods

The study was carried out in compliance with local research ethics committee (LREC) guidelines as a service development.

This was a retrospective study. Forty formalin-fixed paraffin-embedded (FFPE) samples were obtained from patients diagnosed with gliomas (10 oligodendroglial tumours and 30 glioblastomas) from the archives of the Cellular Pathology department at St George’s Hospital, Tooting. The oligodendroglial tumours were from 2005 to 2010 and the glioblastomas were all from 2011. A haematoxylin and eosin (H&E) stained section from each sample was reviewed to ensure the specimen comprised sufficient tumour for diagnosis and further testing.

### Immunohistochemistry

Immunohistochemistry (IHC) staining was carried out on 4 μm sections heated for 30 min at 60°C using Bond III fully automated staining system with their Bond Polymer Refine detection system and associated reagents supplied by Leica Microsystems, Newcastle-Upon-Tyne, UK.

Antigen retrieval and dilution was carried out according to antibody: CD34 clone QBEND10 (Novocastra, Leica Microsystems, UK) 1:100, CD45 clone 2B11&PD7/26 (DakoCytomation, Ely, UK) 1:500, MGMT clone 3.1 (Millipore, Thermo Fisher, UK) 1:100 with Epitope Retrieval Solution 1 (pH6) at 100°C for 30 min, CD68 clone 514H12 (Novocastra, Leica Microsystems, UK) 1:100 with Epitope Retrieval Solution 2 (pH9) at 100°C for 20 min. All primary antibodies were applied to the section for 15 min except MGMT that was applied for 30 min. The MGMT antibody was optimized using tonsil as a positive control. Endothelial cell staining also acted as an internal positive control.

Double-labelling utilized a sequential IHC staining method, incorporating a “cocktail” of CD34, CD45 and CD68 as the first antibody visualized with the Bond Polymer Red Refine detection system (supplied by Leica Microsystems, Newcastle-upon-Tyne, UK) and MGMT clone 3.1 as the second antibody.

### Methylation-specific multiplex ligand-dependent probe amplification (MS-MLPA)

Ten x 10 μm sections were used for DNA extraction and subsequent MS-MLPA testing. The area of tumour was manually micro-dissected to enrich the sample. DNA extraction was performed using an in-house method using heat for de-waxing and Chelex-100 and sodium acetate for DNA purification. The probe mix was prepared by MRC Holland (Amsterdam, The Netherlands) and the procedure was carried out according to the guidelines supplied. MS-MLPA was performed on the Beckman Coulter CEQ 8000. DNA yields were quantified using a NanoDrop (Thermo-Scientific, USA).

A methylation ratio was produced by using an average methylation from 4 analysed probes. In fact 6 probes are provided in the kit but the manufacturers have recently recommended that only 4 are used because 2 of the probes are not particularly informative. A result of either methylated or unmethylated was obtained for the MS-MLPA test. A ratio of less than or equal to 0.25 was used as a cut-off for unmethylated cases in accordance with Jeuken *et al*. [10]. It was also possible, according to Jeuken *et al*. [10] to subcategorise the results as follows: 0 to 0.25 unmethylated, 0.26 to 0.50 mildly methylated, 0.51 to 0.75 moderately methylated and 0.76 to 1.00 extensively methylated. The analysis was performed using the software program Coffalyser v7 (MRC Holland, Netherlands).

### Photography

The slides were viewed on a Nikon Eclipse 80i microscope with a Nikon DS Ri1 12 megapixel camera attached. Images were captured with NIS-Elements BR 3.2 software on a Dell precision T7500 PC. All of the images were captured under standard conditions of illumination. Tiff images (1280 ×1024 pixels) were taken at an objective lens magnification of 40×. At the outset of the session, part of the slide with no tissue (blankfield) was viewed in the microscope and a white balance carried out. The level of illumination was monitored as being between 204 and 206 for the blankfield (between photographs the illumination level was checked as being within the same range). Photographs were taken with a fixed exposure time and after using the inbuilt focusing device.

### Validation and scoring

Quantitation of the labelling index for single-labelled MGMT (number of stained nuclei divided by total number of nuclei expressed as percentage) was carried out in the software program imageJ version 1.46e from the National Institute of Health (Rasband, W.S., http://rsbweb.nih.gov/ij/). Three photomicrographs were taken by the neuropathologist (LB) as representative of the tumour, avoiding non-informative areas such as cortex and necrosis and without knowledge of the methylation status. We use the ImmunoRatio plug-in, which has been validated for the assessment of the labelling index of nuclear markers in breast cancer [19]. In our material we found good correlation of the labelling index for MGMT single-labelling obtained using ImageJ and ImmunoRatio compared to a direct cell count (Pearson correlation coefficient r = 0 .90) and a semiquantitative manual assessment by group of observers (r = 0.80) in 11 oligodendroglial tumours [20]. Please note that one of these cases was omitted from our final study due to insufficient tissue.

Scoring of the double-labelling was carried out from photomicrographs. Slides were photographed “blind” (i.e. without knowledge of the methylation status) by the neuropathologist (LB) and the resulting 120 images (three representative images from each of 40 cases) were evaluated by LB and by two laboratory scientists EB and KE. The scheme followed was that images with mainly negative staining in tumour cell nuclei scored 0, images with equivocal staining scored 1 and images with mainly positive staining scored 2. The final score for each observer was the modal average of their scores for the three images. One observer (LB) carried out a second set of scores after 72 hours. Inter-observer and intra-observer kappa statistics were calculated using the attribute agreement analysis function in the statistical program Minitab 16. Photomicrographs were used to standardise the comparison bearing in mind the different skill-sets of the assessors (please note however that LB works directly from the double-labelled glass slides in everyday diagnosis).

Specificity, sensitivity, positive predictive value and negative predictive value were calculated according to standard formulae in comparison with the gold-standard MLPA after cross-tabulating the results in Minitab 16. For this, the tripartite double-labelling scores were converted to a binary scoring system (0 and 1). Original scores of 0 and 1 were mapped to 1 in the new system and original scores of 2 were mapped to 0. This "reverse" scoring was adopted in order to correspond to the scoring of the MLPA (i.e. unmethylated = 0, methylated = 1). After due consideration, we adopted a convention by which MGMT-equivocal immunostaining was interpreted as MGMT-negative (i.e. presumptive of a methylated status; please see Results).

We wondered whether the "cocktail" (CD34, CD45 and CD68) used in the double-labelling gave a comparable result to the component antigens used singly. Using a thresholding method in image J we measured the area fraction of staining in 10 oligodendroglial tumours for the cocktail and for CD34, CD45 and CD68 stained singly. There was good correlation between the area fraction for the cocktail (range 3 to 8%) and the sum of the area fractions for CD34, CD45 and CD68 stained singly (range 3 to 9%; Spearman's rho = 0 .76). The area fraction of the cocktail was somewhat lower than the combined area fractions of the single labels consistent with a degree of overlap in antigenicity of target cells (e.g. CD68-positive macrophages may also stain with CD45).

## Competing interests

The authors declare that they have no competing interests.

## Authors’ contributions

EB to study design and immunohistochemistry. MG to study design and molecular genetics. KE to study design and immunohistochemistry. FB to molecular genetics. RT to study design and molecular genetics. MC to clinical aspects. LRB to study design, image analysis, statistics and drafting of manuscript. All authors read and approved the final manuscript.

## References

[B1] Gorlia T van den Bent MJ Hegi ME Mirimanoff RO Weller M Cairncross JG Eisenhauer E Belanger K Brandes AA Allgeier A Nomograms for predicting survival of patients with newly diagnosed glioblastoma: prognostic factor analysis of EORTC and NCIC trial 26981-22981/CE.3 Lancet Oncol 2008 1 29 38 10.1016/S1470-2045(07)70384-4 18082451

[B2] Hegi ME Diserens AC Godard S Dietrich PY Regli L Ostermann S Otten P Van MG De TN Stupp R Clinical trial substantiates the predictive value of O-6-methylguanine-DNA methyltransferase promoter methylation in glioblastoma patients treated with temozolomide Clin Cancer Res 2004 1 1871 1874 10.1158/1078-0432.CCR-03-0384 15041700

[B3] Herrlinger U Rieger J Koch D Loeser S Blaschke B Kortmann RD Steinbach JP Hundsberger T Wick W Meyermann R Phase II trial of lomustine plus temozolomide chemotherapy in addition to radiotherapy in newly diagnosed glioblastoma: UKT-03 J Clin Oncol 2006 1 4412 4417 10.1200/JCO.2006.06.9104 16983109

[B4] Stupp R Hegi ME Mason WP van den Bent MJ Taphoorn MJ Janzer RC Ludwin SK Allgeier A Fisher B Belanger K Effects of radiotherapy with concomitant and adjuvant temozolomide versus radiotherapy alone on survival in glioblastoma in a randomised phase III study: 5-year analysis of the EORTC-NCIC trial Lancet Oncol 2009 1 459 466 10.1016/S1470-2045(09)70025-7 19269895

[B5] Quillien V Lavenu A Karayan-Tapon L Carpentier C Labussiere M Lesimple T Chinot O Wager M Honnorat J Saikali S Comparative assessment of 5 methods (methylation-specific polymerase chain reaction, MethyLight, pyrosequencing, methylation-sensitive high-resolution melting, and immunohistochemistry) to analyze O6-methylguanine-DNA-methyltranferase in a series of 100 glioblastoma patients Cancer 2012 1 4201 4211 10.1002/cncr.27392 22294349

[B6] Preusser M Charles JR Felsberg J Reifenberger G Hamou MF Diserens AC Stupp R Gorlia T Marosi C Heinzl H Anti-O6-methylguanine-methyltransferase (MGMT) immunohistochemistry in glioblastoma multiforme: observer variability and lack of association with patient survival impede its use as clinical biomarker Brain Pathol 2008 1 520 532 18400046 10.1111/j.1750-3639.2008.00153.x PMC8095504

[B7] Nakasu S Fukami T Baba K Matsuda M Immunohistochemical study for O6-methylguanine-DNA methyltransferase in the non-neoplastic and neoplastic components of gliomas J Neurooncol 2004 1 333 340 10.1007/s11060-004-9170-6 15662974

[B8] Stupp R Hegi ME Methylguanine methyltransferase testing in glioblastoma: when and how? J Clin Oncol 2007 1 1459 1460 10.1200/JCO.2006.09.7139 17442986

[B9] Watanabe R Nakasu Y Tashiro H Mitsuya K Ito I Nakasu S Nakajima T O6-methylguanine DNA methyltransferase expression in tumor cells predicts outcome of radiotherapy plus concomitant and adjuvant temozolomide therapy in patients with primary glioblastoma Brain Tumor Pathol 2011 1 127 135 10.1007/s10014-011-0022-8 21331613

[B10] Jeuken JW Cornelissen SJ Vriezen M Dekkers MM Errami A Sijben A Boots-Sprenger SH Wesseling P MS-MLPA: an attractive alternative laboratory assay for robust, reliable, and semiquantitative detection of MGMT promoter hypermethylation in gliomas Lab Invest 2007 1 1055 1065 10.1038/labinvest.3700664 17700563

[B11] Brell M Tortosa A Verger E Gil JM Vinolas N Villa S Acebes JJ Caral L Pujol T Ferrer I Prognostic significance of O6-methylguanine-DNA methyltransferase determined by promoter hypermethylation and immunohistochemical expression in anaplastic gliomas Clin Cancer Res 2005 1 5167 5174 10.1158/1078-0432.CCR-05-0230 16033832

[B12] Kreth S Thon N Eigenbrod S Lutz J Ledderose C Egensperger R Tonn JC Kretzschmar HA Hinske LC Kreth FW O-methylguanine-DNA methyltransferase (MGMT) mRNA expression predicts outcome in malignant glioma independent of MGMT promoter methylation PLoS One 2011 1 e17156 10.1371/journal.pone.0017156 21365007 PMC3041820

[B13] Kreth S Limbeck E Hinske LC Schutz SV Thon N Hoefig K Egensperger R Kreth FW In human glioblastomas transcript elongation by alternative polyadenylation and miRNA targeting is a potent mechanism of MGMT silencing Acta Neuropathol 2013 1 671 681 10.1007/s00401-013-1081-1 23340988

[B14] Uno M Oba-Shinjo SM Camargo AA Moura RP Aguiar PH Cabrera HN Begnami M Rosemberg S Teixeira MJ Marie SK Correlation of MGMT promoter methylation status with gene and protein expression levels in glioblastoma Clinics (Sao Paulo) 2011 1 1747 1755 10.1590/S1807-59322011001000013 22012047 PMC3180167

[B15] Pollack IF Hamilton RL Sobol RW Burnham J Yates AJ Holmes EJ Zhou T Finlay JL O6-methylguanine-DNA methyltransferase expression strongly correlates with outcome in childhood malignant gliomas: results from the CCG-945 Cohort J Clin Oncol 2006 1 3431 3437 10.1200/JCO.2006.05.7265 16849758

[B16] Lalezari S Chou AP Tran A Solis OE Khanlou N Chen W Li S Carrillo JA Chowdhury R Selfridge J Combined analysis of O6-methylguanine-DNA methyltransferase protein expression and promoter methylation provides optimized prognostication of glioblastoma outcome Neuro Oncol 2013 1 370 381 10.1093/neuonc/nos308 23328811 PMC3578486

[B17] Cao VT Jung TY Jung S Jin SG Moon KS Kim IY Kang SS Park CS Lee KH Chae HJ The correlation and prognostic significance of MGMT promoter methylation and MGMT protein in glioblastomas Neurosurgery 2009 1 866 875 10.1227/01.NEU.0000357325.90347.A1 19834398

[B18] Ingold B Schraml P Heppner FL Moch H Homogeneous MGMT immunoreactivity correlates with an unmethylated MGMT promoter status in brain metastases of various solid tumors PLoS One 2009 1 e4775 10.1371/journal.pone.0004775 19274096 PMC2652028

[B19] Tuominen VJ Ruotoistenmaki S Viitanen A Jumppanen M Isola J ImmunoRatio: a publicly available web application for quantitative image analysis of estrogen receptor (ER), progesterone receptor (PR), and Ki-67 Breast Cancer Res 2010 1 R56 10.1186/bcr2615 20663194 PMC2949645

[B20] Burke E Grobler M Elderfield K Butler S Taylor R Crocker M Bridges LR Computerized image analysis in the assessment of MGMT immunostaining Neuropathol Appl Neurobiol 2012 1 Suppl. 1 30 31

[B21] Chinot OL Barrie M Fuentes S Eudes N Lancelot S Metellus P Muracciole X Braguer D Ouafik L Martin PM Correlation between O6-methylguanine-DNA methyltransferase and survival in inoperable newly diagnosed glioblastoma patients treated with neoadjuvant temozolomide J Clin Oncol 2007 1 1470 1475 10.1200/JCO.2006.07.4807 17442989

[B22] Capper D Mittelbronn M Meyermann R Schittenhelm J Pitfalls in the assessment of MGMT expression and in its correlation with survival in diffuse astrocytomas: proposal of a feasible immunohistochemical approach Acta Neuropathol 2008 1 249 259 10.1007/s00401-007-0310-x 17965865

[B23] Rodriguez FJ Thibodeau SN Jenkins RB Schowalter KV Caron BL O'Neill BP James CD Passe S Slezak J Giannini C MGMT immunohistochemical expression and promoter methylation in human glioblastoma Appl Immunohistochem Mol Morphol 2008 1 59 65 18091318 10.1097/PAI.0b013e31802fac2f

[B24] Karayan-Tapon L Quillien V Guilhot J Wager M Fromont G Saikali S Etcheverry A Hamlat A Loussouarn D Campion L Prognostic value of O6-methylguanine-DNA methyltransferase status in glioblastoma patients, assessed by five different methods J Neurooncol 2010 1 311 322 10.1007/s11060-009-0031-1 19841865

